# Interfrontal Bone Among Inbred Strains of Mice and QTL Mapping

**DOI:** 10.3389/fgene.2019.00291

**Published:** 2019-04-02

**Authors:** Heather Zimmerman, Zhaoyu Yin, Fei Zou, Eric T. Everett

**Affiliations:** ^1^Dental Research, School of Dentistry, The University of North Carolina at Chapel Hill, Chapel Hill, NC, United States; ^2^Department of Biostatistics, Gillings School of Global Public Health, The University of North Carolina at Chapel Hill, Chapel Hill, NC, United States; ^3^Department of Pediatric Dentistry, School of Dentistry, The University of North Carolina at Chapel Hill, Chapel Hill, NC, United States

**Keywords:** inbred mouse strains, interfrontal bone, wormian bone, QTL, quantitative trait, skeletal variant

## Abstract

The interfrontal bone (IF) is a minor skeletal trait residing between the frontal bones. IF is considered a quasi-continuous trait. Genetic and environmental factors appear to play roles in its development. The mechanism(s) underlying IF bone development are poorly understood. We sought to survey inbred strains of mice for the prevalence of IF and to perform QTL mapping studies. Archived mouse skulls from a mouse phenome project (MPP) were available for this study. 27 inbred strains were investigated with 6–20 mice examined for each strain. Skulls were viewed dorsally and the IF measured using a zoom stereomicroscope equipped with a calibrated reticle. A two generation cross between C3H/HeJ and C57BL/6J mice was performed to generate a panel of 468 F2 mice. F2 mice were phenotyped for presence or absence of IF bone and among mice with the IF bone maximum widths and lengths were measured. F2 mice were genotyped for 573 SNP markers informative between the two strains and subjected to linkage map construction and interval QTL mapping. Results: Strain dependent differences in the prevalence of IF bones were observed. Overall, 77.8% or 21/27, of the inbred strains examined had IF bones. Six strains (C3H/HeJ, MOLF/EiJ, NZW/LacJ, SPRET/EiJ, SWR/J, and WSB/EiJ) lack IF bones. Among the strains with IF bones, the prevalence ranged from 100% for C57BL/6J, C57/LJ, CBA/J, and NZB/B1NJ and down to 5% for strains such as CAST/Ei. QTL mapping for IF bone length and widths identifies for each trait one strong QTL detected on chromosome 14 along with several other significant QTLs on chromosomes 3, 4, 7, and 11. Strain dependent differences in IF will facilitate investigation of genetic factors contributing to IF development. IF bone formation may be a model to understand intrasutural bone formation.

## Introduction

Variations of minor skeletal traits among mice have been reported (Grüneberg, 1952, 1955; Searle, 1954; Deol, 1958). These variant skeletal traits may involve the axial or appendicular skeleton as well as the craniofacial region. Regarding the latter, the interfrontal bone (IF) has been a described skeletal variant (Keeler, 1933; Truslove, 1952; Johnson, 1976). The IF when present resides within the interfrontal (metopic) suture, often in the anterior region near the nasal bones. The IF bone is described as a quasi-continuous trait in that it is either present or not (dichotomous) and when present exhibits morphological variability. Crosses between inbred strains suggest that the basis of the IF to be as a complex trait involving multiple genes (Grüneberg, 1955). Among inbred strains studied earlier C57BL6 and CBA consistently exhibit the IF bone (Truslove, 1952). Abnormal IF bone morphology is present in a number of classical mouse mutants including brain hernia (*bh/bh*), fidget (*fi/fi*), short face (*Pfas^Sofa^*), and short head (*sho/sho*) as examples (Fitch, 1961; Johnson, 1976; Palmer et al., 2016). Development of the IF bone can be also influenced by mutations affecting neural tube development, i.e., *Gli3* (*Xt^bph^*) and *Zic3* (*Zic3^Bn^*) (Johnson, 1969, 1976; Carrel et al., 2000). More recently KO of fibulin-1 leads to reduction in both frontal and IFs (Cooley et al., 2014). Also, perturbation of interfontal suture closure through an Ambn-Msx2 axis leads to thinning and widening of IF bones in mice (Atsawasuwan et al., 2013). The aim of this study is to survey inbred strains of mice for the prevalence of IF and to perform QTL mapping studies with the goal to later understand the genetic factors that determine IF bone formation. To that end a better understanding of IF bone formation may lead to understanding more about other intrasutural bone formation, e.g., wormian bones, as well as aspects ofcranial suture biology.

## Materials and Methods

### Mice

The survey of the prevalence of the IF bone across inbred strains utilized archived skulls from the Mouse Phenome Project (MPP): Collaborations Program sponsored by The Jackson Laboratory to ETE. Strain selection was based upon criteria for participation in the MPP Collaborations Program. All mice were provided by The Jackson Laboratory (Bar Harbor, ME, United States). A total 27 inbred strains were investigated and included: 129S1/SvImJ, A/J, AKR/J, BALB/cByJ, C3H/HeJ, C57BL/10J, C57BL/6J, C57/LJ, C58/J, CAST/Ei, CBA/J, DBA/1J, DBA/2J, DBA/LacJ, FVB/NJ, LP/J, MOLF/EiJ, NOD/LtJ, NZB/B1NJ, NZW/LacJ, PERA/EiJ, PL/J, SJL/J, SM/J, SPRET/Ei, SWR/J, and WSB/Ei. For strains DBA/1J, LP/J, NZW/LacJ, and WSB/EiJ only male skulls were available for phenotyping and for strains SPRET/EI, and DBA/Lac only female skulls were available for phenotyping. Three to ten male and female mice for each strain were examined. Mice were 48–64 days of age (57 + 4 days) accessioned and assigned a unique randomized identification numbers. There was no period of acclimatization. Upon receipt or within 24 h each mouse was euthanized using CO2 gas and weighed (+0.1 g). Body weights along with date of birth and age (days) at the time of euthanasia were recorded. All animal work for the survey of strains was performed under Indiana University School of Dentistry Animal Care and Use Committee (IACUC) approval.

For the QTL mapping, F2 were generated from crosses performed between parental strains (B6C3HF1/J; Stock No. 100010) The Jackson Laboratory. A panel of 468 mice was generated. Mice were euthanized for IF bone measurements at 50–52 days of age. All animal work for the QTL mapping was performed under University of North Carolina at Chapel Hill Animal Care and Use Committee (IACUC) approval.

### Preparation of Skulls

Following euthanasia, the heads are removed, cleaned of skin, fur, loose musculature, and the tongue is removed. The head is then soaked in 1–2% sodium hypochlorite (5.25% sodium hypochlorite diluted in 0.9% NaCl) for 14–16 h at room temperature. The cleaned skulls are thoroughly rinsed in fresh 0.9% NaCl and allowed to air dry (2–3 days) prior to varnishing with a clear polyurethane spray.

### IF Bone Measurements

Skulls were viewed dorsally and the IF measured using a zoom stereomicroscope equipped with a calibrated reticle. Triplicate direct measurements of maximum IF widths and lengths were made on each animal.

### Histology

A head from an adult C57BL/6J mouse was fixed in 10% NBF. A portion of the head was dissected and embedded in paraffin for sectioning. Slides were stained with haematoxylin and eosin using standard methods.

### Genomic DNA Preparation

For each F2 animal the liver and spleen were snap frozen in liquid N2 and stored in a 3.6 ml cryovial at -80°C. Genomic DNA was prepared using the Gentra Puregene Tissue Kit (QIAGEN, Germantown, MD, United States) and stored in ddH2O. Each F2 genomic DNA was quantitated by nano-drop and then 1 mcg of genomic DNA was run on a 0.8% TAE agarose gel and stained to assess possible degradation. 4 μg of DNA was diluted to 100 ng/ul in water and frozen was submitted for genotyping.

### Genotyping

A total of 468 F2 samples were genotyped on the mapping and developmental analysis panel (MMDAP; Partners HealthCare Center for Personalized Genetic Medicine, Cambridge, MA, United States). The MMDAP contained 748 SNP markers of which 573 SNP markers were informative between the B6 and C3H strains and passed our quality control check with <20% missing genotypes. The genotype QC process involved merging datasets and recoding alleles into the special formats for QTL mapping using R/qtl. In addition non-informative parental alleles were not considered and would include SNPs with missing rate >20%. A few markers, found on chromosomes 4, 5, 7, and 10 showed unusual patterns where the genetic distances among markers are too large to be proper. Typically the middle marker will cause such problems. This could be due to genotyping error, or data entry error or transmission distortion, etc. We further examined those markers SNP 6245715 (chr5), SNP 3703981 (chr4), SNP 07-074-764 (chr7), and SNP 6394370 (chr10) with phenotype variables using Fisher’s exact test and ANOVA. Those few markers were not considered in the QTL mapping.

### QTL Mapping

Linkage map construction and interval QTL mapping were performed using R/QTL^1^ (Broman et al., 2003). For phenotype variables “ave_length” and “ave_width” there were many zeros (absence of IF bones). The two part model in R/qtl was applied and is more appropriate in modeling such zero inflated traits (Boyartchuk et al., 2001; Broman et al., 2003). Statistical significance was evaluated by the empirical permutation procedure (Churchill and Doerge, 1994) and for each trait, total 1000 permutation was performed to estimate the 95 and 90% thresholds.

### Statistics

For phenotyping descriptive statistics (mean and standard deviation) were calculated from triplicate measurements of the IF bones from individual animals. The strain/sex group means, standard deviations, and medians were calculated using PASW Statistics version 18.0.0 (SPSS Inc., Chicago, IL, United States). One-way analysis of variance (ANOVA) was performed when comparing the means between males and females within strains. Differences were considered significant when *p* < 0.05.

## Results

### Survey of Interfrontal Bones Among Inbred Mouse Strains

**FIGURE 1:**
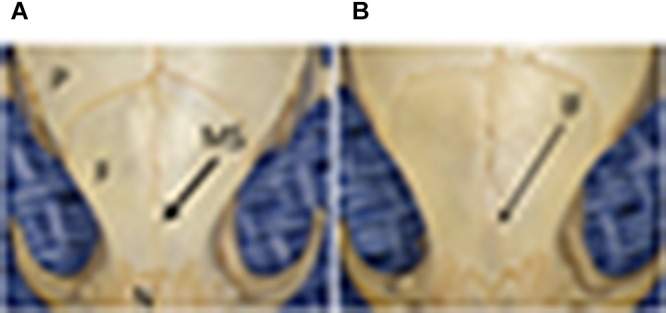
Stereozoom images of a CAST/EiJ mouse **(A)** and a CBA/J mouse **(B)** skulls showing represented absence and presence of the IF bone. P, parietal bone; F, frontal bone; N, nasal bone; MS, metopic/interfrontal suture; IF, interfrontal bone.

**FIGURE 2:**
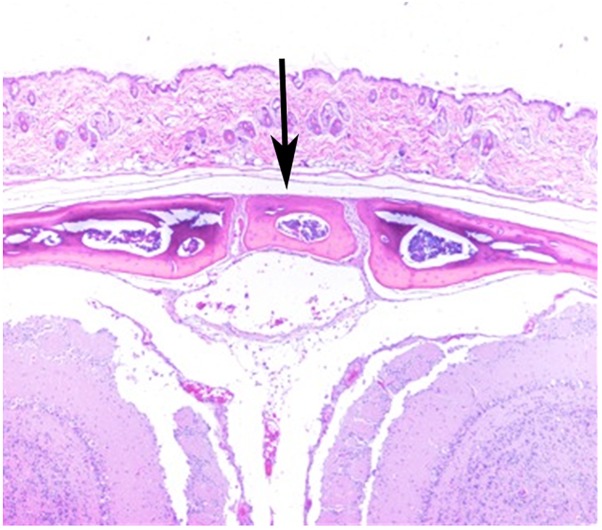
H&E of a coronal section from a C57BL/6J mouse showing IF (arrow).

Stereozoom images of a CAST/EiJ mouse (Figure 1A) and a CBA/J mouse (Figure 1B) skulls showing represented absence and presence of the IF bone. Representative IF histology (Figure 2) shows a coronal section from an adult C57BL/6J mouse. The IF bone resides as a well demarcated bone surrounded by fibrous connective tissue within the interfrontal suture. Also shown is a hematopoietic island within the IF bone.

**FIGURE 3:**
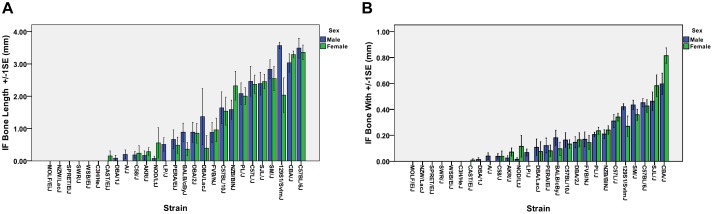
Mean IF bone lengths and widths across all strains. **(A)** IF bone lengths and **(B)** IF bone widths. Male, blue boxes and females green boxes.

Strain dependent differences in the prevalence of IF bones were observed (Table 1). Overall, 77.8% or 21/27, of the inbred strains examined had IF bones. Six strains of mice (C3H/HeJ, MOLF/EiJ, NZW/LacJ, SPRET/EiJ, SWR/J, and WSB/EiJ) lack IF bones. Among the strains that possessed IF bones, the prevalence ranged from 100% for strains such as C57BL/6J, C57/LJ, CBA/J, and NZB/B1NJ down to 5% for strains such as CAST/Ei. For each mouse strain, the mean, median, and standard deviation was calculated for the IF bone width and length. The mean IF bone widths and lengths for each strain is shown in Figure 3A,B, respectively. Across all strains there were no significant differences in IF bone length (*p* = 0.970) or width (*p* = 0.498) between males and females. After comparing males and females within each strain, three strains (129S1/SvImJ, C58/J, and PL/J) demonstrated significant differences in IF bone lengths between males and females (*p* = 0.011, *p* = 0.045, and *p* = 0.021, respectively) and in two strains (CBA/J and C58/J) IF bone widths differed between males and females (*p* = 0.022 and 0.040, respectively). We considered differences to be significant when *p* < 0.05. IF bone lengths and widths appear as correlated traits (Figure 3).

**Table 1:** Survey of Interfrontal bones (IFs) among inbred mouse strains^a^.

Strain	No. of mice with IF/total No of mice examined^b^	% Mice with IF	Strain	No. of mice with IF/total No. of mice examined	% Mice with IF
129S1/SvImJ	17/20	80	DBA/LacJ	3/6	50
A/J	2/20	10	FVB/NJ	12/20	60
AKR/J	8/30	27	LP/J	9/14	64
BALB/cByJ	9/20	45	MOLF/Ei	0/20	0
C3H/HeJ	0/20	0	NOD/LtJ	3/23	13
C57BL/6J	20/20	100	NZB/B1NJ	20/20	100
C57BL/10J	14/20	70	NZW/LacJ	0/14	0
C57/LJ	20/20	100	PERA/Ei	7/20	35
C58/J	4/20	20	PL/J	19/20	95
CAST/Ei	1/20	5	SJL/J	19/20	95
CBA/J	20/20	100	SM/J	19/20	95
DBA/2J	12/20	60	SPRET/Ei	0/8	0
DBA/1J	1/6	17	SWR/J	0/20	0
			WSB/EiJ	0/10	0


*^a^Dried skulls from inbred mouse strains used in the study. All mice were originally obtained from The Jackson Laboratory (Bar Harbor, ME, United States) and then dried skulls prepared by bleaching. Strain selection was based upon criteria for participation in the Mouse Phenome Project Collaborations Program; ^*b*^Male and female mice combined, strains NOD, SPRET/EI, and DBA/Lac had only female skulls*.

### Phenotyping of the B6xC3H F2 Panel

Two breeding pairs (B6C3HF1 mice, *N* = 4) that were retired were examined. All had IF bones and the mean IF bone length 1.03 + 0.74 mm and width 0.48 + 0.16 mm and were generally smaller than that seen in the parental C57BL/6J animals, mean IF bone length 3.42 + 0.81 and width 0.44 + 0.13 mm.

When examining the entire F2 panel (*N* = 468) male and female F2 mice were considered together. 35.3% of all F2s lacked an IF. The remaining F2s showed variability for IF bone lengths and widths. Separating F2s based upon sex, 21.3% F2 males lacked IF bones, whereas 49.9% F2 females lacked IF bones. Sex differences for IF bone was significant (*p* < 0.001) for IF bone mean length (2.01 ±1.39 mm) and mean width (0.30 ±0.23 mm) which are greater in male F2 mice than female F2 mice 1.09 ±1.29 mm and 0.13 ±0.16 mm, respectively.

### QTL Mapping

**FIGURE 4:**
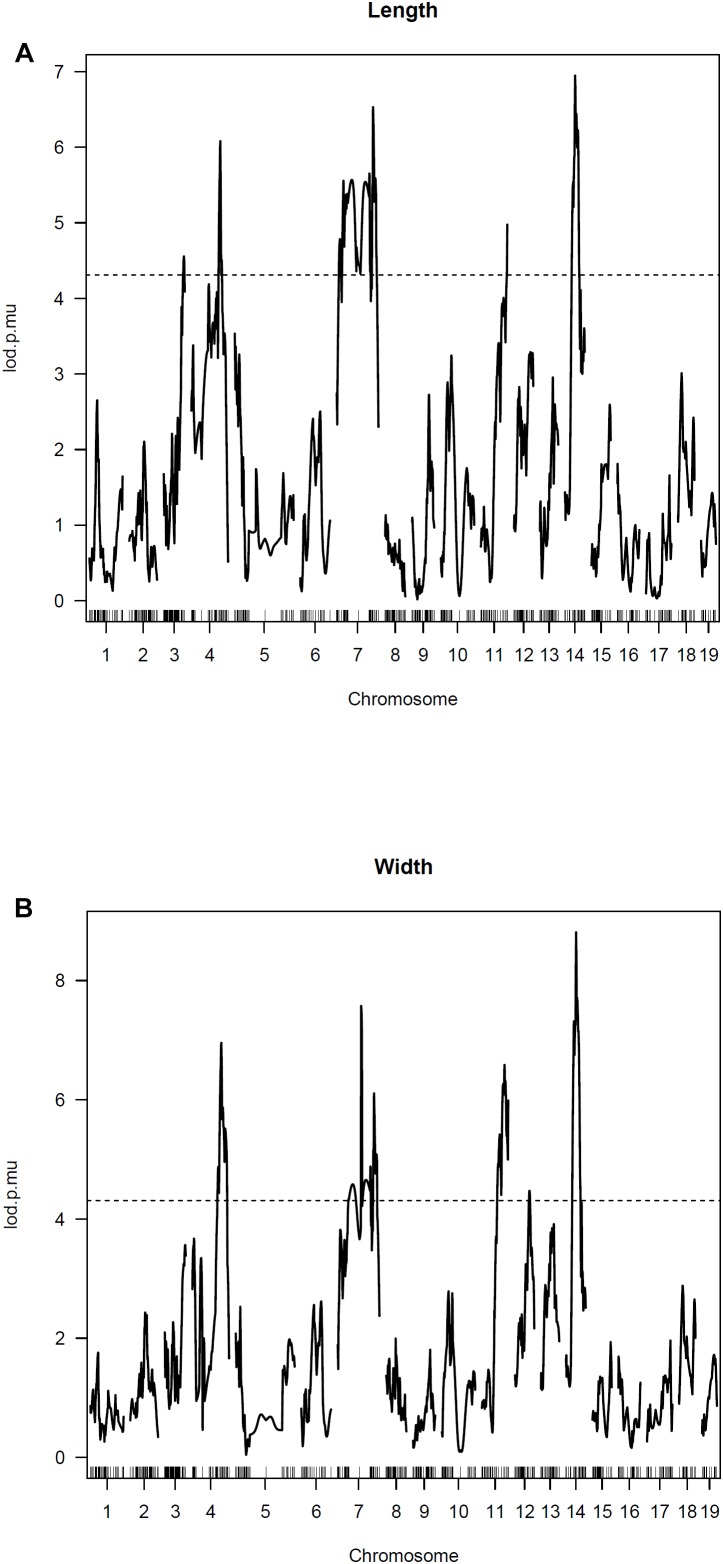
Genomewide scan for IF bone phenotypes. Two part single QTL model was used. LOD score curves for IF bone length **(A)** and width **(B)**.

For IF bone length and width, one strong QTL is detected on Chromosome 14 along with several other significant QTLs on Chromosomes 3, 4, 7, and 11 (Figure 4). The LOD score curves for IF bone width and length are very similar. The two traits appear highly correlated and are likely affected by some common genes. Summary of the peak LOD scores and marker locations are shown in Table 2.

**Table 2:** QTL mapping of IF.

	Marker	Chromosome	Marker position (cM)	Marker position (bp)^a^	Peak LOD score^b^
Average length	c3.loc75	3	75.0		4.20
	gnf04.119.329 (rs27567417)	4	68.5	123501395/123999192	5.82
	rs6322316	7	61.9	120484415/120693266	5.78
	rs6346368	11	99.4	119851494/120035454	4.57
	rs3699179	14	38.2	60723374/61769264	7.21
Average width	gnf04.119.329 (rs27567417)	4	68.5	123501395/123999192	6.95
	rs6322316	7	61.9	120484415/120693266	5.18
	rs6346368	11	99.4	119851494/120035454	5.44
	rs3699179	14	38.2	60723374/61769264	9.03


*^a^Build 36 position/Ensembl *Mus musculus* version 93.38 (GRCm38.p6) position; ^*b*^Maximum LOD scores for peaks above the 5% threshold for phenotypes*.

## Discussion

This study sought to determine among different inbred strains of mice the presence of IF bones. The selection of inbred strains was to represent genetic diversity and to mirror those strains commonly used in the MPP (Paigen and Eppig, 2000; Grubb et al., 2014). Among the inbred strains examined, the presence of the IF bone is consistent with being a quasi-continuous trait in that it is either present or not (dichotomous) and when present exhibits phenotypic variability. The presence of the IF bone among the strains examined can loosely fall into three groups, strains that either do not demonstrate the IF bone or show the IF bone as an infrequent trait (0–20%); strains that demonstrate a wide variation in the prevalence of IF bones (20–80%); and strains that typically have high occurrence of IF bones (80–100%). Acknowledging that mouse strains can change over several decades, we found the high IF bone frequency among CBA/J, C57BL were somewhat consistent with historical observations (Truslove, 1952; Johnson, 1976; Fukuta et al., 1988). Similarly for BALB/cByJ as a strain with less frequent presentation of IF bones (Fukuta et al., 1988). The survey of strains was somewhat limited in representing both sexes in that for three strains (NOD, SPRET/EI, and DBA/Lac) we had access to only female skulls. Sexual dimorphism for size of the IF bone was observed for a few inbred strains. However, it remains unclear whether sexual dimorphism in the size of the IF could simply reflect sexual dimorphism in overall size, since overall size was not reported.

The selection of the C57BL/6J and C3H/HeJ strains for QTL mapping was based upon each strain falling within the extreme state of having or not having IF bones. We found that IF bone lengths and widths were highly correlated traits. Such that IF bones with greater lengths tended to have greater widths. This observation of IF bone lengths and widths being highly correlated traits was also evident in the peak LOD values being associated with the same markers on chromosomes 3, 4, 7, and 11.

The origin of IF bones remains obscure. However, IF bones may resemble other intrasutural bones such as wormian bones. As such, a better understanding of the genetic and environmental factors that influence IF bone development may be relevant to understanding wormian bone formation. Wormian bones (also known as sutural bones or ossicles) are small irregular bones that are present within sutures or fontanelles. Wormian bones can be idiopathic, present as a minor skeletal variant or can occur with numerous recognized syndromes (Gorlin et al., 2001). Wormian bones are often found in osteogenesis imperfectas (Cremin et al., 1982; Semler et al., 2010), cleidocranial dysplasia (Mundlos, 1999) as well as other bone dysplasias (Langer et al., 1991; Horovitz et al., 1995; Santolaya et al., 1998; Garavelli et al., 2009; Megarbane et al., 2014; Palav et al., 2014). The occurrence of wormian bones is thought to be the result of disturbed osteogenesis/ossification or as a response to mechanical forces affecting sutures (Sanchez-Lara et al., 2007; Bellary et al., 2013). The bregmatic bone, a type of wormian bone, occupying the anterior fontanelle, has been reported in at least one case where metopic synostosis was presented (Stotland et al., 2012). Wormian bones are also associated with metopism, (Cirpan et al., 2016). The occurence of wormian bones in the normal population has not been clearly defined. However, they remain a recognized skeletal variant that may be present more often than thought (Hauser and De Stefano, 1989; Marti et al., 2013).

The IF bone as a minor skeletal trait stimulates interest that may be broader in significance relating to metopic suture biology. In humans and other mammals the metopic suture fuses early, typically in the posterior region near the junction of the coronal sutures. Disturbances in this process can lead to premature fusion (trigonalcephaly), which is associated with numerous recognized syndromes or with persistent metopism. *Gli3* loss of function leads to premature closure of the interfrontal suture in mice (Veistinen et al., 2012). Perturbation of interfontal suture closure through the Ambn-Msx2 axis leads to thinning and widening of IF bones in mice (Atsawasuwan et al., 2013). In mice, the region where the IF bone forms corresponds to the glabella in humans. Disturbances in the glabella can lead to encephaloceles and other nasio-cranial problems. Anatomically, the location of IF bones may have bearing on a number of conditions affecting humans. A better understanding of IF bone formation may lead to understanding more about other intrasutural bone formation, e.g., wormian bones, as well as aspects of cranial suture biology.

## Ethics Statement

All animal work for the survey of strains was performed under Indiana University School of Dentistry Animal Care and Use Committee (IACUC) approval. All animal work for the QTL mapping was performed under University of North Carolina at Chapel Hill Animal Care and Use Committee (IACUC) approval.

## Author Contributions

EE, HZ, and FZ contributed to conception and design of the study. EE, ZY, and FZ organized the database. ZY, FZ, and EE performed the statistical analysis. HZ and EE wrote the first draft of the manuscript. HZ, EE, and FZ wrote sections of the manuscript.

## Conflict of Interest Statement

The authors declare that the research was conducted in the absence of any commercial or financial relationships that could be construed as a potential conflict of interest.

## Footnotes

1 http://www.rqtl.org
